# Rare Case of Delayed Bleeding Occurring 8 Years After Percutaneous Nephrolithotomy and Angioembolization: A Case Report and Current Literature Review

**DOI:** 10.3390/reports9020135

**Published:** 2026-04-27

**Authors:** Răzvan Alexandru Dănău, Răzvan-Ionuț Popescu, Aida Petca, Viorel Jinga, Răzvan-Cosmin Petca

**Affiliations:** 1 Department of Urology, “Carol Davila” University of Medicine and Pharmacy, 8 Eroii Sanitari Blvd., 050474 Bucharest, Romania; razvan.danau@umfcd.ro (R.A.D.); viorel.jinga@umfcd.ro (V.J.); razvan.petca@umfcd.ro (R.-C.P.); 2 Department of Urology, “Prof. Dr. Th. Burghele” Clinical Hospital, 20 Panduri Str., 050659 Bucharest, Romania; 3 Department of Obstetrics and Gynecology, CF2 Clinical Hospital, 63 Marasti Blvd., 011464 Bucharest, Romania

**Keywords:** PCNL, angioembolization, hematuria, arteriovenous fistula, pseudoaneurysm

## Abstract

Background and Clinical Significance: Over recent decades, percutaneous nephrolithotomy (PCNL) has emerged as a primary treatment, firmly establishing itself as the cornerstone approach for managing large kidney stones. Postoperative bleeding commonly stems from an arteriovenous fistula (AVF), a connection between a damaged artery with high flow and a damaged vein with low flow, or from a pseudoaneurysm (PA), which involves arterial blood leaking into the tissue, causing a localized hematoma. The preferred technique for addressing such vascular complications is selective trans-arterial angioembolization, widely regarded as the gold standard. Case Presentation: In this article, we present the case of a 42-year-old woman who experienced delayed bleeding eight years after PCNL and a previous angioembolization. The patient presented with macroscopic hematuria, and further investigations, including cystoscopy, contrast-enhanced abdominal-pelvic CT, and angiography, were performed. To stop the bleeding, we identified and performed selective angioembolization (SAE) of a small arterial branch arising from an inferior branch of the right renal artery. Conclusions: To the best of our knowledge, this is the initial documented instance of delayed bleeding manifesting eight years post-PCNL and angioembolization. This occurrence is exceptionally rare, given that the patient exhibited no urological signs or symptoms over the intervening years, and no predictive or risk factors were identified.

## 1. Introduction and Clinical Significance

Percutaneous nephrolithotomy (PCNL) has emerged as a predominant therapeutic modality over the past few decades, establishing itself as the cornerstone intervention for managing sizable renal calculi [1]. Its ascendancy to this position is primarily attributed to its high efficacy in extracting large and complex renal calculi [2].

Despite the relatively high level of procedural safety, it is not without complications, with hemorrhage being the most commonly encountered. Depending on the circumstances, hemorrhage may manifest intraoperatively or postoperatively (early or delayed) [2]. The most prevalent causes of postoperative bleeding are arteriovenous fistula—AVF (a communication established between an injured high-flow artery and an injured low-flow vein) and pseudoaneurysm—PA (arterial blood leakage within the parenchyma resulting in a localized hematoma) [3,4,5,6,7]. Gross hematuria is almost invariably the most important clinical sign. Significant hemorrhage may manifest during the early postoperative phase (within 2–14 days following PCNL), while AVF formation typically presents as a delayed complication approximately 3–6 weeks after PCNL [8].

The preferred technique for addressing such vascular complications is selective trans-arterial angioembolization, widely regarded as the gold standard [4]. Nonetheless, in numerous instances, conservative management alone proves adequate for cessation of bleeding (embolization for severe post-PCNL hemorrhage is required in less than 2% of patients) [2,4]. Although angioembolization is the most efficient method to cease the bleeding, special conditions such as contrast hypersensitivity and renal insufficiency should not be ignored. In these circumstances, it becomes crucial to explore novel methods to control and stop immediate postoperative hemorrhage, such as tamponade on the nephrostomy drainage in post-PCNL, when used [9].

This case report aims to present a rare case of a patient who experienced delayed postoperative hemorrhage eight years following PCNL and angioembolization.

## 2. Case Presentation

A 42-year-old female was admitted to Prof. Dr. Th. Burghele Clinical Hospital, one of the high-volume urology centers in Bucharest, Romania, and also a certified Excellence Center for Uro-Lithiasis affiliated with the European Association of Urology, for macroscopic hematuria, which had its onset 3 h before admission, and right flank pain, which altered her general condition. Generalized fatigue, palpitations, and dizziness completed the presentation, which had progressively developed over the last 48 h.

In the patient’s medical history, there is a significant record of multiple surgical interventions for right renal lithiasis. Twelve years ago, an imaging examination revealed a calculus in the right ureteropelvic junction, for which Extracorporeal Shock Wave Lithotripsy (ESWL) was performed. A second round of ESWL was performed 9 years ago. One year later, the patient was diagnosed with right renal lithiasis (approximately 20 mm calculus located in the renal pelvis), leading to Percutaneous Nephrolithotomy (PCNL). According to the medical records, the procedure was performed in the prone position; the puncture site was the lower calyx, and Alken dilators were used to access the pyelocaliceal system. Postoperatively, at 48 h, the patient experienced intermittent macroscopic hematuria and suspicion of arteriovenous fistula at the level of the right kidney, prompting selective angioembolization with two VortX Diamond© coils. Between 2016 and 2024, the patient experienced no urological disorders. Beyond this period, she had no other known pathologies or surgical interventions in her medical history. Additionally, the patient had no prescribed or self-administered medications, including anticoagulants or antithrombotics, previous to hospital admission.

The physical examination revealed abdominal pain, more pronounced in the right flank, tenderness, and pallor. Upon admission, the body temperature was assessed to be within the normal range. The cardiovascular and respiratory assessments indicated tachycardia (110 beats/min) without tachypnea (respiratory rate—15 breaths/min). The measured blood pressure was 102/75 mmHg, with an oxygen saturation of 98%.

Complete blood tests were performed, and the results indicated mild leukocytosis (WBC: 12.5 × 10^9^/L) and neutrophilia (NE: 9.4 × 10^9^/L), with normal hemoglobin levels (Hgb: 12.2 g/dL). The evolution of blood tests throughout the hospital stay is illustrated in Table 1. The urine analysis revealed hematuria and leukocytes, while a urine culture was negative.

The routine ultrasound imaging evaluation performed upon admission revealed suspicion of blood clots present in the right renal pelvis, but without any degree of hydronephrosis.

**Figure 1:**
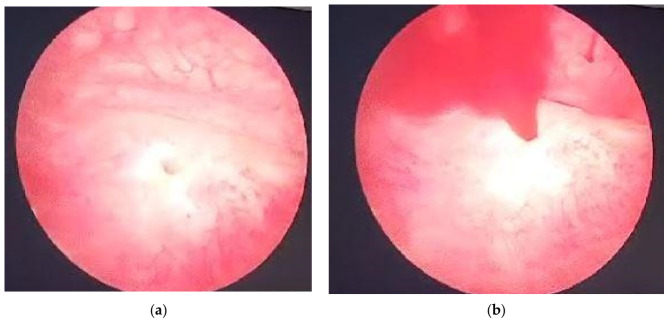
Cystoscopy; (**a**) Right ureteral orifice; (**b**) Spontaneous sanguineous emission at the site of the right ureteral orifice.

Because the hospital design does not include an emergency imagistic department, an immediate CT exploration was not possible at that time. After admission, conservative measures were initiated, including lumbar compression and the administration of Tranexamic Acid. For a quick evaluation of the bleeding source, in the context of a possible aggressive emergency, a cystoscopic evaluation was performed under spinal anesthesia, revealing no discernible source of bleeding within the bladder. Spontaneous sanguineous emission was observed at the right ureteral orifice (Figure 1). Antibiotic prophylaxis was initiated with Ceftazidime 1 g, based on local sensitivity data [10]. During conservative treatment measures, the patient had totally stable vital signs, without forcing surgical or interventional decisions.

**Figure 2:**
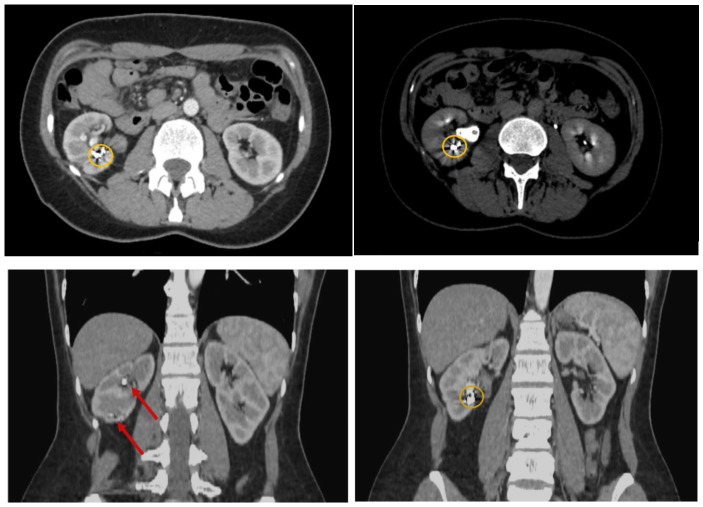
Contrast-enhanced CT (axial and sagittal views) revealing multiple hematic accumulations at various stages of degradation (indicated by orange circles) and lithiasis (indicated by red arrows).

Following cystoscopy, a contrast-enhanced computed tomography was performed on the first morning after admission to evaluate the abdomen and pelvis (Figure 2). The CT report indicated multiple hematoma collections at various stages of degradation, predominantly recent (with average native densities of approximately 60 Hounsfield Units) within the right renal collecting system, without evidence of dilation. At the lower pole level, multiple pseudoaneurysm-like images were observed. These lesions appeared within the renal sinus, approximately 10 mm in maximum diameter, and were in intimate contact with the lower and middle calyceal stems. A series of metallic structures was identified and described in the report as clips (most probably the coils used during the last angioembolization). A small number of unobstructive stone fragments was also identified, distributed throughout all the calyces, with a maximum diameter of 5 mm.

**Figure 3:**
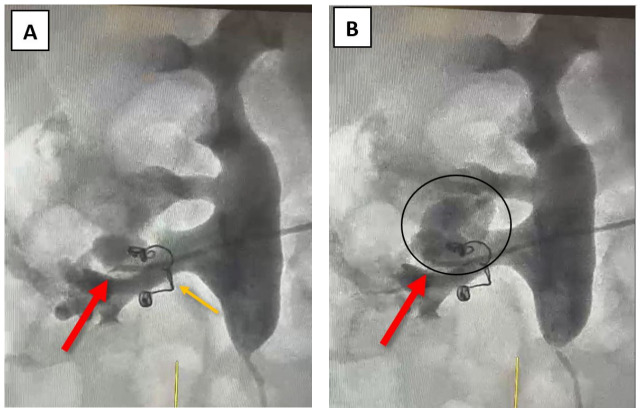
Angiography—(**A**) the source of bleeding is recognizable at the site of a small inferior renal vessel—indicated by the red arrow; (**B**) blush generated by the selective administration of the contrast agent—indicated by the black oval; the orange arrow indicates the two coils that were previously used for the angioembolization.

At this point, a fistula between an arterial vessel and the right renal pelvis was suspected, and further investigations, such as angiography, were recommended to facilitate data correlation. Angiography was performed via the right brachial artery using a Cobra 4F catheter. After selective administration of the contrast agent, the source of bleeding was identified at the site of a small inferior renal arterial vessel (Figure 3A,B). The coils used during the last angioembolization (eight years ago) are shown in Figure 3.

**Figure 4:**
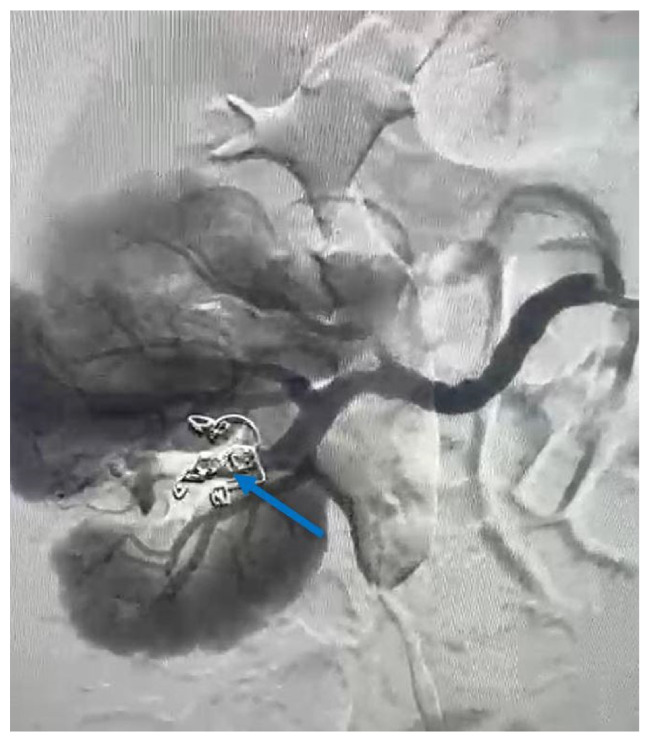
Angiographic aspect of the right kidney after angioembolization (the blue arrow indicates the VortX Diamond© coils, which have been used).

To control and cease the hemorrhage, a guidewire was introduced at the vessel’s site, and selective embolization was carried out using two VortX Diamond© coils of 6/7.7 mm each (Figure 4). The procedure time of the angioembolization was 7 min and 50 s.

Twenty-four hours after the procedure, the patient reported improved general condition and mild lumbar pain (2/10 on the visual analog scale), without hematuria or other physical signs of bleeding. Upon discharge, the patient presented with a good general condition, afebrile, stable hemodynamics, normal skin and mucous membrane coloration, effortless micturition, and clear urine.

## 3. Discussion

Although numerous complications can arise following PCNL, renal hemorrhage remains the most common, with an incidence ranging from 11.2% to 17.5% [8]. As mentioned before, the most common causes of major postsurgical bleeding are represented by vascular complications such as pseudoaneurysms, arteriovenous fistula, and segmental arterial injury [3,4].

A case–control study conducted from April 2015 to April 2018 determined that a history of diabetes and renal anomalies may serve as predictive factors for delayed post-PCNL hemorrhage [2]. Although tubeless PCNL (replacement of the standard postoperatively placed nephrostomy tube with an internal drainage that consists of a double-J stent or a ureteral catheter) significantly revealed major advantages regarding operative time, hospital stay, or postoperative urinary fistulae, the occurrence of severe postoperative hemorrhage is still debatable in various large studies [11,12]. A prospective observational study inferred that arterial hypertension, puncture site, and operative time have a noteworthy impact on estimated hemoglobin deficiency during PCNL [13].

However, angioembolization remains the gold standard for managing these situations, and the risk of complications, including nephrectomy, persists [14,15,16,17]. These complications may also occur after renal biopsy, partial nephrectomy, or urological trauma [4]. Another adverse consequence of super-selective renal angioembolization is functional impairment; however, this effect is more pronounced in patients with solitary kidneys [18]. Associated signs and symptoms, such as hematuria and lumbar pain, should be promptly recognized, and further investigations should be performed.

In the present case, the investigations were initiated with a complete blood count to assess the hemoglobin level and evaluate the severity of the anemia. We continued the investigations with cystoscopy to exclude any specific bleeding source within the bladder. Considering that the source of bleeding is located at the level of the upper urinary tract and considering the patient’s urologic history, we conducted a contrast-enhanced CT scan, which guided us regarding the location of the lesion. In terms of treatment, we decided that angioembolization is the best choice.

Since the second half of the last century, endovascular access has been the most common approach to managing hemorrhagic events in urology [19,20]. Angioembolization presents itself with the advantages of being minimally invasive, safe, suitable even for critical patients, and effective, especially as an emergency procedure, not only in cases of arteriovenous fistula following PCNL, but also in situations like preoperative infarction of renal tumors, treatment of angiomyolipoma, vascular malformations, medical renal disease, and complications following renal transplantation [21,22].

Renal hemorrhage can be controlled with various embolic agents, although some studies report the greater effectiveness of coils in several clinical situations [19,20,23,24,25,26]. The main reasons discussed include the facile manipulation, lower rates, smaller areas of renal ischemia—therefore a lower degree of renal function impairment—and better tracking accuracy of the embolic agent after detachment in the target vessel [19]. As described and pictured earlier in Figure 4, coils were also used in the present case as an embolic agent during the angioembolization.

Vascular access is usually achieved through the femoral artery, but the brachial or axillary arteries represent valid alternatives in cases of femoral artery occlusion [23]. The sheath caliber should be 5 French or larger, depending on the complexity of the procedure. Various types of catheters can be used: Cobra-configured, RC-2-shaped, SOS-shaped, Levi (Lev-1), and Simmons-shaped. The main argument for choosing one type over another rests on the anatomical particularities of the renal arteries and the associated pathologies affecting the vessels [23]. Microcatheters are extremely useful for supraselective angioembolization.

In the setting of AVF, the success of angioembolization is achieved using superselective catheters with coaxial systems and micro coils as the embolic agent. It is highly recommended not to use particles, given the high risk of pulmonary embolism due to passage through the communication between the artery and the vein. The technical goal is achieved when the fistula is completely obliterated, at the cost of minimal impairment of renal function [23].

Lee et al. conducted a retrospective study on 62 patients who underwent renal artery embolization between January 2009 and December 2019 [27]. In this study, it was pointed out that a large number of patients who undergo renal artery angioembolization may have underlying kidney disease or arterial hypertension. It was demonstrated that although no patient developed chronic kidney disease in the short term after the procedure a noticeable decline in the estimated glomerular filtration rate was observed in the long term. The study concluded that, although supraselective angioembolization is most of the time a safe procedure, acute kidney injury still counts as a serious possible complication [27].

Irani et al. conducted a case–control study in which several risk and predictive factors for delayed bleeding after percutaneous nephrolithotomy requiring angioembolization have been proposed and evaluated—history of diabetes, history of arterial hypertension, history of ESWL, history of PCNL, history of open surgery, coagulation disorders and the presence of a renal anomaly—only history of diabetes and the presence of a renal anomaly were found to be statistically significant [2]. According to El-Nahas et al., upper caliceal puncture, solitary kidney, staghorn stone, multiple punctures, and an inexperienced surgeon have all been identified as significant risk factors for severe bleeding [28]. Loo et al. concluded that arterial hypertension, puncture site, and operative duration significantly impact estimated Hb deficiency during PCNL [12]. Other factors that may predict a hemorrhagic event include stone burden, degree of hydronephrosis, urinary tract infection, American Society of Anesthesiologists (ASA) grade, and operative time [29,30,31]. Apart from the history of ESWL, none of the factors mentioned above were present in our patient’s case.

Considering the patient’s medical history, which includes a prior selective angioembolization (SAE), our focus shifted to identifying potential risk factors that may have contributed to or predicted the procedure’s failure. In a study that evaluated the predictive factors of SAE failure for moderate- to high-grade renal trauma, Baboudijian et al. found gross hematuria, hemodynamic instability, and urinary extravasation to be statistically significant [32]. After the first SAE, our patient did not exhibit any of these factors. In a case report published by Seno et al., another set of risk factors was identified as significant in predicting initial super-selective renal arterial embolization failure; this set includes multiple percutaneous access sites, more than two bleeding sites on a renal angiogram, and the use of a gelatin sponge alone as embolic material [3]. In our case, the patient presented with only one percutaneous access site during the initial angioembolization and a single bleeding site on the renal angiogram. During the angioembolization procedure, two coils were used (as depicted in Figure 3—indicated by the orange arrow). Thus, none of the aforementioned situations were present in our patient’s case. The CT scan identified residual fragments with maximum diameters less than 5 mm located in the pyelo-calyceal system, without interfering with the renal parenchyma. These were disposed of only in the collecting system without determining obstruction, and no inflammation or other processes that could have led to bleeding complications were noted. Usually, hematuria caused by residual fragments comes from the injuries provoked by the moving stones. This appears as mild hematuria and does not cause significant blood loss. Arterio-venous fistula repermeabilization was positioned right next to the previous angioembolization deep in the renal parenchyma and may have been caused by collateral vessel formation or a small vessel missed during the first procedure due to vascular spasm [33], but none of these theories was certainly identified during the embolization procedure.

To the best of our knowledge, this is the first reported case of delayed bleeding occurring eight years after PCNL and angioembolization. This case is even rarer considering that the patient exhibited no urological signs or symptoms during the intervening years. The absence of any known risk or predictive factor may serve as a key element for further investigations into other scenarios that could potentially lead to vascular and hemorrhagic complications similar to those described in this case.

## 4. Conclusions

This case report highlights the importance of considering late vascular complications after PCNL and post-PCNL angioembolization in the diagnostic evaluation of delayed hematuria or renal bleeding. Increased clinical awareness may facilitate timely recognition and appropriate management. However, as this observation is based on a single case, it serves as a warning, not as a routine implementation of systematic long-term follow-up for all patients.

## Figures and Tables

**Table 1:** Patient’s Blood Parameters.

	Day 1	Day 2	Day 3	Post Embolization Day 1	Post Embolization Day 2
WBC (10^3^/µL)	12.5	11.5	11.8	16.1	11.5
Hemoglobin (g/dL)	12.2	11.3	10.6	11.1	11.2
Urea (mg/dL)	17	-	-	16	-
Creatinine (mg/dL)	0.64	-	-	0.71	-
GFR (mL/min/1.73 m^2^)	110			107	
Quick Value (s)	12	-	-	-	12
Prothrombin Time (%)	92.1	-	-	-	93.1
INR	1.14	-	-	-	1.11

## Data Availability

Data are contained within the article.

## Abbreviations

The following abbreviations are used in this manuscript:

AVF	Arteriovenous fistula
CT	Computed tomography
ESWL	Extracorporeal Shock Wave Lithotripsy
PA	Pseudoaneurysm
PCNL	Percutaneous nephrolithotomy
SAE	Selective angioembolization
